# A Problem Shared Is a Community Created: Recommendations for Cross-Institutional Collaborations

**DOI:** 10.7191/jeslib.754

**Published:** 2023-12-20

**Authors:** Marla Hertz, Kelsey Badger, Lena Bohman, Lucy Carr Jones, Christine Nieman Hislop, Katy Smith, Hao Ye

**Affiliations:** University of Alabama at Birmingham, Birmingham, AL, USA; The Ohio State University, Columbus, OH, USA; Zucker School of Medicine at Hofstra/Northwell, Hempstead, NY, USA; University of Virginia, Charlottesville, VA, USA; University of Maryland, Baltimore, MD, USA; Saint Louis University, St. Louis, MO, USA; University of Pennsylvania, Philadelphia, PA, USA

## Abstract

Committee work is a requisite job function for many in academia, yet designing a productive collaborative experience often remains a challenge. In this article, we reflect on our experiences as part of a successful cross-institutional working group and describe strategies to improve leadership structure, group dynamics, accountability, and incentives for collaborative projects.

As of January 2023, the National Institutes of Health (NIH) Data Management & Sharing (DMS) Policy requires investigators applying for funding to submit a Data Management and Sharing Plan (DMS Plan) that describes how scientific data will be managed, preserved, and shared. In response to this new policy, a community of more than 30 librarians and other research data professionals convened the Working Group on NIH DMSP Guidance, collaboratively producing comprehensive guidance about the policy for researchers and research support staff. In less than a year, the working group produced glossaries of NIH and data management jargon, an example data management and sharing plan, a directory of existing example plans, checklists for researchers and librarians, and an interactive repository finder.

This group was a successful grassroots effort by contributors with diverse expertise and backgrounds. We discuss practical strategies for each stage of activity throughout the lifecycle of the working group; from recruiting members, designing pathways to encourage participation from busy professionals, structuring the meetings to facilitate progress and productivity, and disseminating final products broadly. We invite fellow librarians, data professionals, and academics to apply and build upon these strategies to tackle cross-institutional challenges.

## Introduction

The sharing of research data has been increasingly mandated as a condition for funding (National Institutes of Health 2003; National Science Foundation, n.d.), or publication (Springer Nature, n.d.; PLOS 2019; SAGE publications 2018), as tax-payer supported entities promote public access to the results of federally funded research (Holdren 2013; Nelson 2022). The National Institutes of Health furthered their commitment to “accelerate[s] biomedical research discovery…by enabling validation of research results, providing accessibility to high-value datasets, and promoting data reuse for future research studies” via the NIH Data Management and Sharing (DMS) Policy (National Institutes of Health 2020a). Effective as of January 23, 2023, the DMS Policy requires that NIH-funded researchers submit a two-page Data Management and Sharing (DMS) Plan as part of the funding proposal.

Release of the DMS Policy and supplementary information on the required six elements (National Institutes of Health 2020c), allowable costs (National Institutes of Health 2020b), and repository selection (National Institutes of Health 2020d) sparked concerns about implementation at many universities, especially within research support units like academic libraries. To meet such implementation needs, a grassroots working group of librarians and research data professionals collaborated to develop guidance and resources that would benefit researchers and—particularly—librarians. Formed in June 2022, the Working Group on NIH DMSP Guidance met regularly both as a whole and in subgroups. By December 2022, the working group published a publicly accessible toolkit of resources on the Open Science Framework (OSF, https://osf.io/uadxr (Badger et al. 2022)). The products initially included: two policy readiness checklists for librarians and researchers respectively; multiple glossaries of terms related to data management, grants, and specifically the NIH DMS Policy; and example DMS plans. The outputs expanded to include an extended reference guide in January 2023 and an interactive data repository finder the following April.

Responses to cross-institutional challenges benefit from—if not require—collaborations among contributors with diverse backgrounds and expertise. The individuals of the working group were able to efficiently set and reach objectives that provided value to the broader research community, evidenced by the number of visits and linkbacks to the toolkit. Herein, we describe how our working group came together, coordinated efforts, actualized outcomes, and reviewed progress. We share our reflections as a list of recommendations on the process so that others may learn from, apply, and build upon our foundational experience.

## Group Formation – Open By Design

Inspired by virtual collaboration efforts in the open science community, Hao Ye issued a call for librarians and research support professionals to work together to create educational resources about the NIH DMS Policy.

### Recommendation: Issue a broad call for participants

Volunteers were solicited via listservs from the Data Caucus of the Medical Library Association as well as the Research Data Access & Preservation (RDAP) Association to engage potential contributors from a variety of disciplines and institutions. Participants in the working group included representatives from large and small state universities, private universities, hospitals, nonprofit institutions, and government agencies. There were individuals with deep knowledge of the grant landscape and biomedical research, as well as novices with fresh perspectives. This range of institutional perspectives and levels of expertise informed our approach as we aimed to create resources that could be used at many different kinds of institutions by individuals at varying levels of familiarity with the NIH grant seeking process.

### Recommendation: Engage stakeholders early

An added benefit of the open call for working group participants was the engagement of multiple stakeholder organizations. Representatives of the RDAP executive board as well as staff from the National Center for Data Services (NCDS), a division of the Network of the National Library of Medicine, responded to Ye with interest in supporting the project. After the working group formed, Ye applied for funding from both organizations to support honoraria for contributors and group leaders. In addition to providing funds, RDAP agreed to host the working group’s files on its Google Drive, and NCDS would later promote the group’s resources on their website, including hosting the repository finder application and donating developer time to launch and maintain it. The early commitments of RDAP and NCDS helped formalize and legitimize the working group’s efforts.

## Coordination – Make It Easy To Say Yes

Making it easy to contribute should be considered a baseline for effective group efforts (Nosek 2019). The successful recruitment of nearly three dozen participants presented certain challenges to coordination, including scheduling meetings and agreeing on expectations for engagement. The key to managing these logistics was to take a “just enough” approach to coordination that centered the needs of contributors and made it easy to say yes.

### Recommendation: Share the burden of leadership

Ad hoc groups can suffer from a leadership void that leaves participants uncertain about who is responsible for facilitating meetings and making decisions. In addition to Ye’s continuing role as a facilitator, the working group adopted a rotating leadership model that allowed volunteers to commit to terms of only one month. Responsibilities during this time included preparing the agenda and facilitating meetings, sharing email updates with the group, and responding to participant questions. Additionally, decisions about the working group’s vision and scope were made democratically by the group members. By sharing these responsibilities, we reduced the burden that is often associated with leadership roles.

### Recommendation: Offer multiple ways to engage

It was not possible to schedule a single recurring meeting time that would accommodate everyone’s schedules. Instead, to maximize availability, the group met weekly, alternating each week between two meeting times. The combination of frequency and variability allowed most contributors to attend at least once a month. Importantly, there was no required attendance and the monthly facilitators were responsible for ensuring that significant decisions or deadlines were also communicated to the group by email. To reduce possible confusion about the alternating schedule, all emails and meeting invitations included a link to a central landing page for meeting times, Zoom links, and other key working documents. Google Docs was intentionally chosen as the platform for coordinating collaboration efforts and drafting of content because it allowed synchronous contributions from multiple people and was generally a familiar platform that minimized training or other technical barriers.

### Recommendation: Incentivize participation

We recognized the need for working group participants to justify their time and effort. Thus, we intentionally cultivated incentives through authorship and citation policies (discussed and agreed upon by the group), collaborating to develop letters of recognition for participation, and creating mechanisms for capturing site visits and download analytics from OSF (https://github.com/example-DMS-plans/osf-metrics). This supporting documentation was stored alongside the group products to ensure easy access. These actions both acknowledge effort and support colleagues whose professional evaluation criteria (including tenure) require demonstration of impact.

## Operation – Do The Thing!

The switch from planning to execution is a key turning point in any project. Our approach utilized structured communication and documentation practices to guide the work. At the same time, we embraced flexibility in resource development—groups focusing on separate products had agency to continually refine their objectives to address the overarching goal. This balance of flexibility anchored in a stable framework made it possible to do the thing without going off course.

### Recommendation: Embed accountability

Once specific project aims were established through group consensus, we formed subgroups dedicated to developing each resource output. Each subgroup appointed a project lead to monitor progress and communicate updates to the full working group. These updates helped maintain clear scope boundaries to ensure each group was developing distinct end products and could address overlaps when they occurred. For instance, two subgroups merged when it became evident that their agendas aligned and their outputs were interdependent. As we were under pressure to release the resources in advance of the federal policy start date, we established concrete goals and stuck to them.

### Recommendation: Move fast and break things

Given the imminent arrival of the January 2023 implementation deadline and demand for resources, we adopted a “move fast and break things” ethos, prioritizing speed and experimentation. While this may seem contradictory to the methodical coordination efforts mentioned earlier, these approaches actually work in tandem—a set of clear participatory processes for the group enabled prototyping to proceed without having to gain the full group’s approval for each decision. We aimed to release our work in Fall 2022, with the expectation that we would iterate as more information became available from the NIH. This hard deadline stopped us from becoming bogged down in perfecting every detail before release, and instead kept the focus on communicating the information we had available at the time.

### Recommendation: Include failsafes to self-correct

While the use of subgroups enabled swift progress, they also posed a risk of becoming siloed from the larger working group. Furthermore, the pressure to adhere to tight deadlines increased the likelihood of errors and inconsistencies. To mitigate these challenges, we implemented several failsafes to ensure project success: 1) place a strong emphasis on documentation and establish regular verbal and written communication within the larger group to promptly identify and resolve issues, and 2) instate an internal peer review process for participants of different subgroups to provide feedback on all draft documents, leveraging the collective strengths of the group to ensure quality. This review stage also identified concerns about cohesion and accessibility of the different resources, which were addressed through recommendations in the next section.

## Out Into The World – Dissemination

As resource development neared completion, the working group focused on how to promote the toolkit and engage with those who most needed the resources. The impact of this project was widespread, with over 6,500 page views during the first four months after the toolkit went public. The resources were cross-listed on the websites of more than 50 libraries and other research support organizations, such as the Association of American Medical Colleges.

### Recommendation: Incorporate an accessibility mindset from the beginning

Accessibility considerations arose as documents were revised following peer review. Despite being far into the project when we realized this, it is never too late to adopt an accessible by design mindset. To this end, we prepared a style guide and document template to ensure consistency and accessibility among the final resources. The style guide addressed topics such as standard formatting, font, and spacing, and the inclusion of alternative text for all images or graphics. The final copyedit procedure conformed each resource to our style guide. Learn from our oversight and create a document template with style and accessibility guidelines at the onset of the project to save time in the final editing process.

### Recommendation: Market beyond the library

The toolkit was announced and shared with communities who routinely work with research data, including librarian-facing, researcher-facing and administration-facing groups. Sharing cross-discipline knowledge and resources requires foresight and planning to identify potential users and tailor promotional materials to them. The working group collaborated to disseminate the toolkit through websites, scholarly conferences, webinars, and blog posts to reach a wider audience.

### Recommendation: Learn from each other

This collaboration built professional connections among its members. These connections were especially valuable as many data librarians operate as the sole data services professional at their organization. By working on the toolkit together, members of the working group formed a community for peer learning as we collectively learned the details of this new policy. This experience also provided an opportunity for newer librarians to work with and learn from more experienced practitioners in the field.

## Conclusion

Large-scale collaboration provides broad benefits, but working groups can get mired in detail or lose momentum, ultimately making little progress. Our collaboration was able to produce a widely used toolkit in a relatively short timeframe. This article outlines the elements that made our working group successful. Further policy changes affecting research data management, such as the memo from the White House on Ensuring Free, Immediate, and Equitable Access to Federally Funded Research (also known as the “Nelson memo”), are on the horizon. We intend that these recommendations inspire and facilitate fellow colleagues to launch their own cross-institutional collaborative groups.

Finally, we wish to highlight that collaborative working groups are excellent opportunities for professional development, particularly for early career librarians. Institutions commonly employ either a single research data librarian or a small team, as compared to a large reference department. Thus, data management librarianship can be an isolating profession. As libraries created new data librarian positions in response to the global culture shift towards open science, many of these positions were filled by relatively novice librarians. Our working group was an opportunity to not only build resources to address a new policy, but also a means to learn from each other and build professional connections that extended beyond the bounds of this project.
